# A Study on the C, N, and P Contents and Stoichiometric Characteristics of Forage Leaves Based on Fertilizer-Reconstructed Soil in an Alpine Mining Area

**DOI:** 10.3390/plants12223838

**Published:** 2023-11-13

**Authors:** Yichen Ba, Xilai Li, Yunqiao Ma, Yu Chai, Chengyi Li, Xinyue Ma, Yongxiang Yang

**Affiliations:** 1 College of Agriculture and Animal Husbandry, Qinghai University, Xining 810016, China; 13995174545@163.com (Y.B.); ma_panpan@163.com (Y.M.); chaiyu0731@163.com (Y.C.); chengyi_li0801@163.com (C.L.); 200907010126@qhu.edu.cn (X.M.); 200907010130@qhu.edu.cn (Y.Y.); 2 State Key Laboratory of Plateau Ecology and Agriculture, Qinghai University, Xining 810016, China

**Keywords:** alpine mining area, vegetation type, granular organic fertilizer, sheep manure, nutrient elements, ecological stoichiometric ratio

## Abstract

In this study, we analyzed the C, N, and P contents and stoichiometric characteristics of forage leaves of five species (*Elymus breviaristatus* cv. Tongde, *Poa crymophila* cv. Qinghai, *Puccinellia tenuiflora* cv. Qinghai, *Festuca sinensis* cv. Qinghai, and *Poa pratensis* cv. Qinghai) in “fertilizer-reconstructed soil” through integrative soil amendment with parched sheep manure and granular organic fertilizer in an alpine mining area. A model is fitted in order to screen out the best forage species suitable for vegetation restoration in the alpine mining area and the most favorable fertilizer dosage to improve the nutrient content of forage leaves. The results showed that (1) increasing the dosages of granular organic fertilizer and sheep manure had little effect on the C content of the five types of forage grasses, but they could significantly increase the N and P contents and N/P of the manually restored grassland in the alpine mining area (*p* < 0.05). (2) The productivity and stability of the five species were ranked as follows: *Elymus breviaristatus* cv. Tongde > *Puccinellia tenuiflora* cv. Qinghai > *Festuca sinensis* cv. Qinghai > *Poa pratensis* cv. Qinghai > *Poa crymophila* cv. Qinghai. (3) According to the fitted least squares model and the willingness to maximize the C, N, and P contents of the leaves, the ranking of the five forage grasses was described by the Prediction Profiler as follows: *Elymus breviaristatus* cv. Tongde > *Puccinellia tenuiflora* cv. Qinghai > *Festuca sinensis* cv. Qinghai > *Poa crymophila* cv. Qinghai > *Poa pratensis* cv. Qinghai. (4) The predictive model suggested that the optimal contents of C, N, and P in *Elymus breviaristatus* cv. Tongde, *Festuca sinensis* cv. Qinghai, and *Poa pratensis* cv. Qinghai leaves could be achieved with the application of 3.6 kg/m^2^ of granular organic fertilizer and 45.0 kg/m^2^ of sheep manure. For *Poa crymophila* cv. Qinghai leaves, the ideal content was attained by applying 0 kg/m^2^ of granular organic fertilizer and 45.0 kg/m^2^ of sheep manure. Lastly, the optimal C, N, and P contents in *Puccinellia tenuiflora* cv. Qinghai leaves could be obtained through the application of 3.6 kg/m^2^ of granular organic fertilizer combined with 0 kg/m^2^ of sheep manure. In conclusion, the study’s results highlight the significant practical value of the fertilizer-reconstructed soil for vegetation restoration in alpine mining regions.

## 1. Introduction

Coal mining has made great contributions to the development of society and the global economy, and coal also occupies a fundamental position in China’s energy sector [1,2]. While coal mining has brought considerable economic benefits, it has also tremendously changed the physical and biological environment of mining areas [3]. In particular, open-pit coal mining has resulted in the destruction of surface vegetation, the alteration of microbial communities, water pollution, and reduced biodiversity [4]. The coal gangue hills formed by the accumulation of a large quantity of coal mine debris after a mine is abandoned not only encroach on valuable land resources but also cause destruction to vegetation, water and soil erosion, and serious damage to the local ecological environment [5]. Therefore, effective measures need to be taken to control abandoned mining areas.

The Muli Coalfield in Qinghai of West China forms an important part of the Qilian Mountains’ ecological security barrier [6]. However, Muli lies in the permafrost area of the Qinghai–Tibetan Plateau, a typical ecologically fragile area with harsh environmental conditions. Years of open-pit coal mining operations have accumulated large-scale slag piles, which have seriously damaged the grassland ecosystem. Thus far, it has been reported that vegetation reconstruction in coal gangue hills is an effective ecological restoration measure [7]. Some studies have shown that the coal mine debris matrix formed by weathered coal gangue and permafrost contains sufficient nutrients. After applying a large amount of organic fertilizer, it can meet the initial growth requirements of sown seeds without relying on expensive “alien soil” to reconstruct the coal gangue substrate in the alpine mining area [8]. Jin and Zhang et al. also pointed out that sufficient organic fertilizers need to be supplemented in the early stage of vegetation cultivation. For example, adding sheep manure can not only meet the nutrient requirements of the cultivated vegetation in its early stage of growth in the mining area but also improve the coal gangue substrate of the mining area and increase the microbial diversity of the coal gangue substrate. It is a practical and economic restoration measure for the improvement of mining waste [9,10]. In addition, the environment in the frigid alpine mining areas is harsh, and the low temperature severely restricts the growth and development of cultivated plants [11]. The application of organic fertilizers in very cold mining areas can significantly improve the cold resistance of herbaceous plants [12]. Therefore, the restoration method of using organic fertilizers as a substitute for soil to reconstruct the soil is suitable for highland areas. This study aims to investigate the ideal organic fertilizer dosage for augmenting element content in plant leaves without disrupting plant ecological stoichiometry and to enhance overall primary productivity in high-altitude mining regions.

Ecological stoichiometry is a discipline that studies the interactions and equilibrium of multiple chemical elements in an ecosystem [13]. It has gradually become an important research tool for assessing nutrient utilization efficiency, limiting elements, and global carbon, nitrogen, and phosphorus cycling in ecosystems. It is also a new method for assessing the biological stability of ecosystems [14,15]. The method of ecological stoichiometry was initially utilized to study aquatic ecosystems. Currently, scholars have conducted extensive research on ecological chemometrics in terrestrial ecosystems such as forests, grasslands, and deserts [16,17,18,19]. At present, research on the stoichiometric characteristics of plant organisms in ecologically restored alpine mining areas is relatively lacking. Therefore, understanding the differences in stoichiometric characteristics of plant organisms under different soil nutrient conditions in the ecosystem of alpine mining areas can promote further understanding of nutrient cycles and energy flow in the ecosystem. In addition, fitting regression models is commonly practiced in ecological restoration research, but further optimization and prediction of the restoration outcome through models are commonly used in food science, industrial materials, pharmaceutical formula optimization, and clinical disease risk factor prediction [20,21,22,23]. Few studies have been carried out to study the optimal fertilization ratios for reconstructed soil substrates based on the stoichiometric characteristics of plant C, N, and P. Therefore, this study uses model fitting to predict the optimal fertilization ratio, which can fill the knowledge gap in ecological restoration research in frigid alpine mining areas.

This study focuses on the nutrient dynamics of C, N, and P and on the stoichiometric characteristics of five types of cultivated graminoid forage grasses in the fertilizer-reconstructed soil of an alpine mining area. We intend to establish a predictive model based on the responses of C, N, and P in the leaves of these grasses to different restoration conditions to assess which fertilization ratios can enhance biological productivity and stability the most. By analyzing actual response variables and optimizing the fitted prediction models, the study comprehensively evaluates rational restoration measures in alpine mining areas. This evaluation sheds light on key factors influencing the stability and productivity of the restored plant species, as well as the ranking of pasture performance during the restoration of alpine mining areas. Ultimately, the study aims to provide theoretical insights for restoration projects in such areas.

## 2. Materials and Methods

### 2.1. Overview of the Study Area

**Figure 1:**
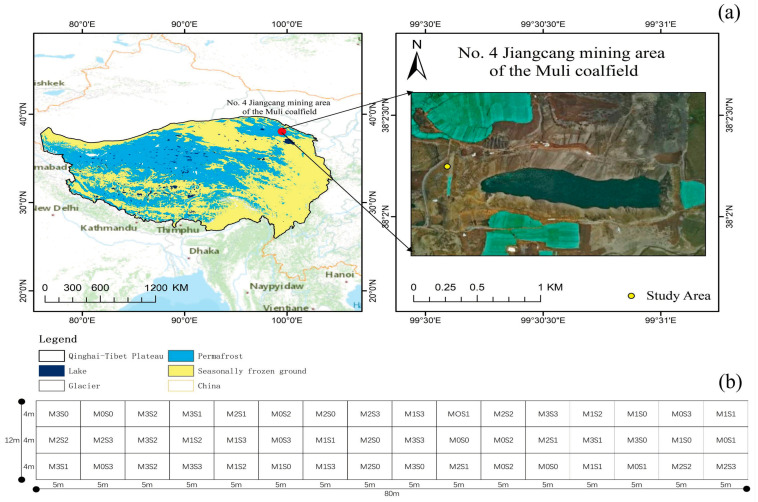
Geographical location (**a**) and layout of sampling plots (**b**).

The Muli Coalfield is located in the Qilian Mountains in the northern Qinghai–Tibetan Plateau, with an area of about 400 km^2^ (Figure 1a). Permafrost is widely distributed in the Muli Coalfield in an island shape with an average thickness of 80 m, but is thicker than 100 m in some areas. The landform is characterized by a high-altitude structural fault-depressed intermountain basin. The Jiangcang mining area is located in the upper reaches of the Datong River in the Muli Coalfield (99°27′~99°35′ E, 38°02′~38°03′ N). The mining area is 25 km long from east to west, 2.5 km wide from north to south, and covers an area of 55 km^2^ (Figure 1a). The altitude is about 3800–4200 m, the annual average temperature is −2.8 °C, the minimum temperature is −35.6 °C, and the maximum temperature is 19.8 °C. The annual precipitation is 477.1 mm, most of which is concentrated in summer, and the annual evaporation is 1049.9 mm. The field samples for this study were collected from the No. 4 pit of the Jiangcang mining area in the Muli Coalfield.

### 2.2. Sampling Design and Sample Collection

#### 2.2.1. Sampling Design

The soil substrate of the sampling site was amended with sheep manure + coal gangue at four ratios of 0:10 (0 m^3^/m^2^, S0), 1.5:10 (0.03 m^3^/m^2^, S1), 3:10 (0.06 m^3^/m^2^, S2), and 4.5:10 (0.09 m^3^/m^2^, S3). The soil matrix was created by mixing equal volumes of coal gangue with corresponding volumes of sheep manure. The amount of sheep manure was applied at four dosages of 0 kg/m^2^ (S0), 15 kg/m^2^ (S1), 30 kg/m^2^ (S2), and 45 kg/m^2^ (S3), respectively. Similarly, the granular organic fertilizer was applied at four levels, 0 kg/m^2^ (M0), 1.2 kg/m^2^ (M1), 2.4 kg/m^2^ (M2), and 3.6 kg/m^2^ (M3), resulting in a total of 16 treatments (M0S0, M0S1, M0S2, M0S3, M1S0, M1S1, M1S2, M1S3, M2S0, M2S1, M2S2, M2S3, M3S0, M3S1, M3S2, and M3S3) involving two types of fertilizers. Each treatment was replicated thrice, culminating in 48 plots, each spanning 4 m × 5 m (refer to Figure 1b).

The physical and chemical properties of the sampling site that was amended with coal gangue, sheep manure, and granular organic fertilizers are provided in Table 1. The properties of the soil matrix formed using fertilizers as the substitute of soil are shown in Table 2.

In May 2021, stones exceeding 5 cm in size were extracted from the sampling site utilizing an excavator, and the reconstituted soil matrix layer was limited to 20 cm thick. Before applying sheep manure, an equivalent volume of coal gangue was removed and replaced with an equal volume of sheep manure and 50% of the intended granular organic fertilizer dosage. Through a combination of excavators, disc harrows, and manual techniques, the parched sheep manure and granular organic fertilizer were mixed. This mixture was then blended with the coal gangue and supplemented by the remaining 50% granular organic fertilizer to form the reconstituted soil matrix. Finally, *Elymus breviaristatus* cv. Tongde, *Poa crymophila* cv. Qinghai, *Puccinellia tenuiflora* cv. Qinghai, *Festuca sinensis* cv. Qinghai, and *Poa pratensis* cv. Qinghai were sown at a rate of 225 kg/hm^2^ along with 225 kg/hm^2^ of specialized pasture fertilizer (total nutrients ≥ 35%, N = 18%, P_2_O_5_ = 12%, and K_2_O = 5%). The sowed ground was prepared for the sampling plot through mechanical and manual methods and then covered with non-woven fabrics.

#### 2.2.2. Sample Collection

In mid-August 2022, during the vigorous growth phase of the plant community, five sub-plots (50 cm × 50 cm) were randomly designated within each sample plot, maintaining a 0.5 m distance from the boundaries. A sufficient quantity of forage leaves representing five types of grasses—*Elymus breviaristatus* cv. Tongde, *Poa crymophila* cv. Qinghai, *Puccinellia tenuiflora* cv. Qinghai, *Festuca sinensis* cv. Qinghai, and *Poa pratensis* cv. Qinghai—was collected. These leaves were chosen based on their complete shape, similar size, and consistent color. The samples were placed into paper bags and transported to the laboratory. Subsequent steps of sample processing included deactivation of enzymes (105 °C, 30 min), followed by further drying to a constant weight (65 °C, 48 h). Afterwards, the samples were ground using a ball mill (ST-M200, Beijing, China), and particles were sieved through a 0.15 mm sieve. Such processed samples were used to determine the C, N, and P contents of forage leaves under distinct fertilization conditions. 

### 2.3. Nutrient Content Determination

The N and P contents of the forage leaves were determined using digestion via concentrated sulfuric acid–hydrogen peroxide, followed by assessment using a continuous flow injection analyzer (AA3-Auto Analyzer III, Bran + luebbe, HH, GER) [24], and the C content of the pasture leaves was determined using the potassium dichromate–H_2_SO_4_ oxidation external heating method [25]. The stoichiometric characteristics of C, N, P, and plants were expressed as a mass ratio.

### 2.4. Data Processing and Statistical Analysis

The test data were organized using Microsoft Excel 2021, while SPSS 26.0 software facilitated the statistical analysis. The K-S method was deployed to assess data normality at the 0.05 level. Differences in the C, N, and P contents and stoichiometric characteristics of the forage leaves were analyzed by a restoration approach and among different species using a one-way ANOVA. Duncan’s method was subsequently used for multiple comparisons at the significance level of 0.05. The influences of sheep manure, granular organic fertilizer, and forage species were investigated through three-way ANOVAs along with their interactions on C, N, and P and the stoichiometry of forage leaves. Least squares regression modeling was used to analyze the effects of varying granular organic fertilizer and sheep manure dosages on the C, N, and P contents of diverse forage leaves so as to predict the optimal fertilization level. The model was fitted based on JMP PRO 16.0.0, and the trend of the C, N, and P contents at different fertilization dosages was compared using the Prediction Profiler. The optimal fertilization ratios for the five pasture types were determined via the Surface Plot Profiler, in which the “maximize” willingness function of the response variable is a three-part piecewise smoothing function that consists of interpolating cubics between the control points and exponentials in the tails. When optimizing the three response variables of C, N, and P, a total willingness function needs to be constructed and optimized. The total willingness of all the willingness is defined as the geometric mean of the individual willingness desirability function [26].

## 3. Results

### 3.1. C, N, and P Contents of Forage Leaves

**Figure 2:**
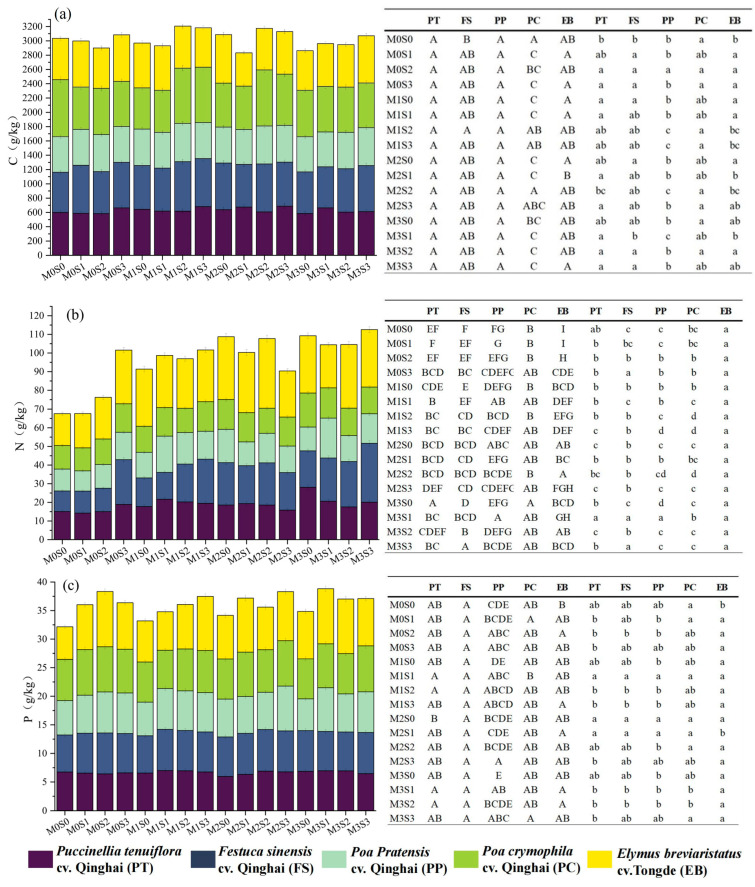
Effects of different fertilizer combinations on C (**a**), N (**b**), and P (**c**) of forage leaves and differences in C (**a**), N (**b**), and P (**c**) contents of different varieties of forage in the same combinations of fertilization treatment. Note: The bar chart shows the C, N, and P contents of five species of forage leaves in 16 fertilization treatments. Different capital letters in the table denote significant differences in nutrients among different treatments for the same species of forage leaves (*p* < 0.05, the same below), and different lowercase letters in the columns denote significant differences in nutrients among different varieties of forage leaf blades in the same fertilization treatment.

Different organic fertilizer dosages have a minimal impact on the C content of the leaves of the five herbaceous species (Figure 2a). Notably, there was no notable difference in the C content among *Puccinellia tenuiflora* cv. Qinghai and *Poa pratensis* cv. Qinghai leaves across the fertilization gradient. Comparatively, *Festuca sinensis* cv. Qinghai displayed a significantly higher C content at the M1S2 fertilization level, outperforming the control, M0S0 (*p* < 0.05). Particularly noteworthy is the peaking of the C content in non-fertilized *Poa crymophila* cv. Qinghai leaves (M0S0), surpassing the lowest C content in the M1S0 treatment by 28%. In turn, *Elymus breviaristatus* cv. Tongde registered its highest C content (M2S0), outperforming the lowest M2S1 treatment by 27%.

As presented in Figure 2b, the highest N content in *Puccinellia tenuiflora* cv. Qinghai and *Poa crymophila* cv. Qinghai leaves occurred in the M3S0 treatment. For *Festuca sinensis* cv. Qinghai, *Poa crymophila* cv. Qinghai, and *Elymus breviaristatus* cv. Tongde, their peak N content manifested in the joint treatments of M3S3, M3S1, and M2S2, respectively. Across the board, the five herbaceous species exhibited the lowest N content at the organic fertilizer dosage of 0 kg/m^2^ (M0). Among them, *Puccinellia tenuiflora* cv. Qinghai, *Poa crymophila* cv. Qinghai, and *Poa pratensis* cv. Qinghai had the lowest N content in M0S1, while *Festuca sinensis* cv. Qinghai and *Elymus breviaristatus* cv. Tongde brevis showcased the lowest in M0S0. Generally, the N content exhibited an upward trend with increasing organic fertilizer dosage. Importantly, changes in sheep manure dosage had a limited impact on the N content compared to the granular organic fertilizer dosage.

Among the five forage grasses, *Poa pratensis* cv. Qinghai showed a substantial difference in the P content among different combinations of fertilizer dosage (*p* < 0.05), whereas the other four forage grasses were less responsive to distinct fertilization levels. Notably, the P content of *Poa pratensis* cv. Qinghai, *Poa crymophila* cv. Qinghai, and *Elymus breviaristatus* cv. Tongde leaves dwindled with reduced granular organic fertilization levels. In essence, different combinations of fertilization levels had the most pronounced effect on forage leaf N content, a significant impact on the forage leaf P content of some species, and a relatively modest effect on the C content.

In the M0S0 treatment, *Elymus breviaristatus* cv. Tongde leaves exhibited the highest N content (Figure 2b), a significant increase of 37% compared to *Festuca sinensis* cv. Qinghai leaves with the lowest N content (*p* < 0.05). *Poa crymophila* cv. Qinghai displayed the highest P and C contents among the five herbaceous species, with notable differences in the C content from the other species (*p* < 0.05). Notably, *Elymus breviaristatus* cv. Tongde had the lowest P content at 5.74 g/kg among the five species. In the context of M0S1, *Elymus breviaristatus* cv. Tongde leaves had the highest N content, while *Festuca sinensis* cv. Qinghai demonstrated the highest C content among the five species. In this fertilization regimen, *Elymus breviaristatus* cv. Tongde showed the highest N and P contents in leaves, significantly differing from the other four species (*p* < 0.05). Meanwhile, *Poa pratensis* cv. Qinghai exhibited the lowest C content, differing significantly from the other species (*p* < 0.05). In the M0S3 treatment, *Puccinellia tenuiflora* cv. Qinghai displayed the highest C, N, and P contents among the five species. In contrast, *Poa pratensis* cv. Qinghai exhibited the lowest C content, significantly differing from the other species (*p* < 0.05). The M1S0 treatment resulted in *Elymus breviaristatus* cv. Tongde leaves having the highest N and P contents, significantly differing from other species (*p* < 0.05). Conversely, *Poa pratensis* cv. Qinghai leaves exhibited the lowest C, N, and P contents in this fertilization regimen (*p* < 0.05). While the N and P contents in *Elymus breviaristatus* cv. Tongde leaves were minimally impacted by the combined fertilization level, their C content displayed a declining trend with increasing granular organic fertilizer dosage.

### 3.2. Ecological Stoichiometric Characteristics of C, N, and P in Forage Leaves 

**Figure 3:**
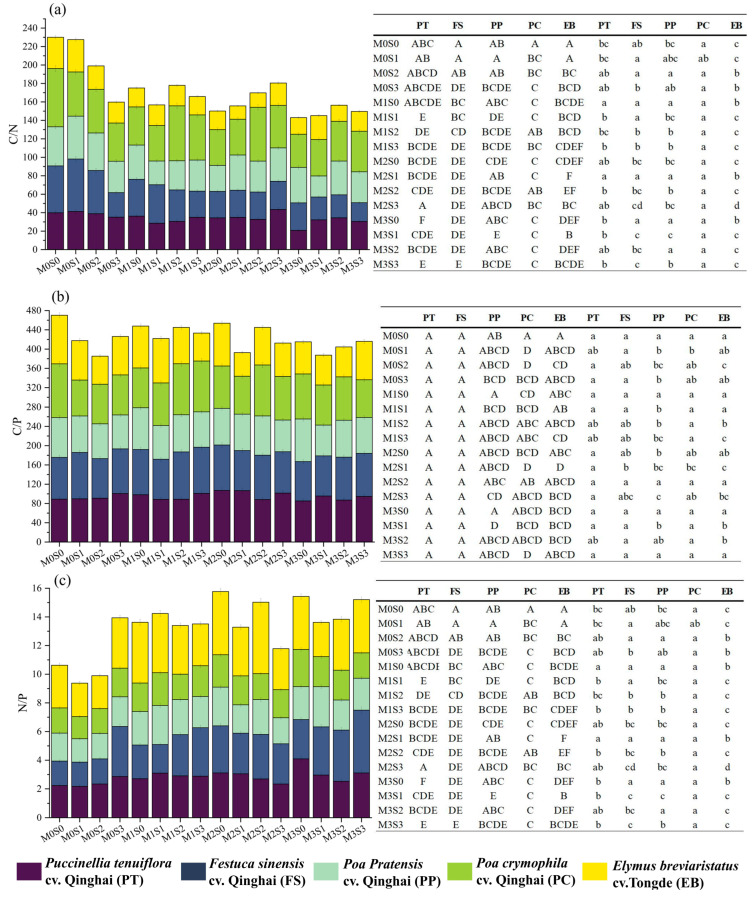
Effects of different fertilizer combinations on the stoichiometric ratio of forage leaves and differences in the stoichiometric ratio of different species of forage grasses in the same fertilizer combination, C/N (**a**); C/P (**b**); N/P (**c**). Note: The bar chart shows the stoichiometric ratio of five species of forage leaves in the 16 fertilization treatments. Different capital letters in the table denote significant differences in the stoichiometric ratio among different treatments for the same species of forage leaves (*p* < 0.05, the same below), and different lowercase letters in the columns denote significant differences in the stoichiometric ratio among different varieties of forage leaf blades in the same fertilization treatment.

As shown in Figure 3a, *Puccinellia tenuiflora* cv. Qinghai exhibited a maximal C/N ratio in the M2S3 treatment but a minimal ratio in M3S0, differing significantly from the M3S3 treatment (*p* < 0.05). For *Festuca sinensis* cv. Qinghai, *Poa pratensis* cv. Qinghai, and *Elymus breviaristatus* cv. Tongde leaves, their highest C/N ratio occurred at a single sheep manure dosage of 15 kg/m^2^, while *Festuca sinensis* cv. Qinghai’s minimal C/N value of 20.42 materialized at the highest dosages of sheep manure and granular organic fertilizer (M3S3). In *Poa pratensis* cv. Qinghai, the lowest C/N ratio was seen in the M3S1 treatment. *Poa crymophila* cv. Qinghai exhibited its highest C/N ratio without organic fertilizer treatment, reaching 64.06. At the maximal dosage of organic fertilizer, C/N dropped to its lowest value of 36.22.

No significant differences in leaf C/P emerged between *Puccinellia tenuiflora* cv. Qinghai and *Festuca sinensis* cv. Qinghai among different fertilization treatments. The leaves of *Poa crymophila* cv. Qinghai demonstrated the highest C/P and N/P when treated with 3.6 kg/m^2^ of granular organic fertilizers (M3S0), while the lowest C/P appeared in the M3S2 treatment, significantly differing from the M3S0 treatment (*p* < 0.05).

The highest N/P in *Festuca sinensis* cv. Qinghai leaves was evidently associated with the treatment of the highest dosage of sheep manure and granular organic fertilizer (M3S3). Conversely, the lowest N/P transpired in the unfertilized treatment (M0S0), showcasing a significant difference (*p* < 0.05). *Puccinellia tenuiflora* cv. Qinghai, *Festuca sinensis* cv. Qinghai, *Poa pratensis* cv. Qinghai, and *Poa crymophila* cv. Qinghai all reached their maximum N/P values in M3, which were 4.11, 4.44, 2.81, and 2.60, respectively. The smallest leaf N/P across the five forage grasses occurred in the M0S1 treatment. Generally, the N/P trend was positively correlated with the amount of organic fertilizer applied.

In the M0S0 treatment, *Poa crymophila* cv. Qinghai leaves displayed the highest C/N ratio (Figure 3), significantly exceeding the lowest C/N (*p* < 0.05) of *Elymus breviaristatus* cv. Tongde leaves. *Elymus breviaristatus* cv. Tongde leaves achieved a maximum N/P ratio of 3.06, significantly surpassing that of other forage leaves (*p* < 0.05). Among M0S0, M0S1, M0S2, and M0S3 treatments, *Elymus breviaristatus* cv. Tongde leaves consistently exhibited the lowest C/N values among the five species. In these treatments, *Poa crymophila* cv. Qinghai consistently held the highest leaf C/N ratio. Of the five species, *Elymus breviaristatus* cv. Tongde registered the highest leaf N/P ratio in all sheep manure dosage treatments. Moreover, in the three single organic fertilizer dosage treatments, *Elymus breviaristatus* cv. Tongde leaves showcased the highest leaf N/P ratio. Overall, leaf C/P had a minimal variation with forage variety in identical fertilization treatments. Among the five species, *Elymus breviaristatus* cv. Tongde, the large-grained forage grass, consistently exhibited the largest leaf N/P ratio in all 16 treatments.

### 3.3. Analysis of Organic Fertilizer Dosage and Forage Species Interactions

#### 3.3.1. Three-Way ANOVA

The outcomes of the three-way ANOVA (Table 3) revealed that interactions involving granular organic fertilizer, dosage levels, grass species, and sheep manure dosage exerted significant effects on grass leaf C content. The C content varied significantly with vegetation type (*p* < 0.01), the impact of which was also notable (*p* < 0.05). Vegetation type, granular organic fertilizer dosage, sheep manure dosage, and their mutual interactions all significantly influenced the N content (*p* < 0.01). Additionally, vegetation type and sheep manure dosage exerted a significant impact on the P content as well.

The application of sheep manure alone had no effect on leaf C/N, while other factors and their interactions significantly impacted leaf C/N (*p* < 0.01). The interaction of vegetation and granular organic fertilizer dosage, along with the interactions among forage grass species, granular organic fertilizer dosage, and sheep manure dosage, held no significant sway over leaf C/P. However, forage type varied significantly with leaf C/P (*p* < 0.01), while the remaining factors had a significant influence (*p* < 0.05). Leaf N/P was notably affected by sheep manure dosage, whereas other factors and their interactions exhibited a strong impact on leaf N/P (*p* < 0.05).

#### 3.3.2. Analysis of Significant Factors Affecting Fertilizer Dosage

**Figure 4:**
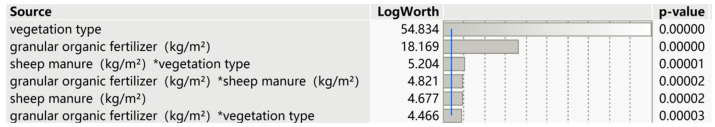
Summary and ranking of various factors affecting the C, N, and P contents of forage leaves. Note: *p* < 0.01 can be determined to be a highly significant factor, with larger *p*-values indicating less effects. LogWorth is defined as −log10(*p*-value), and LogWorth values above 2 are significant at the 0.01 level (−log10(0.01) = 2). This transformation adjusts the size of the *p*-value for subsequent plotting. “*” is the interaction between the variables.

**Figure 5:**
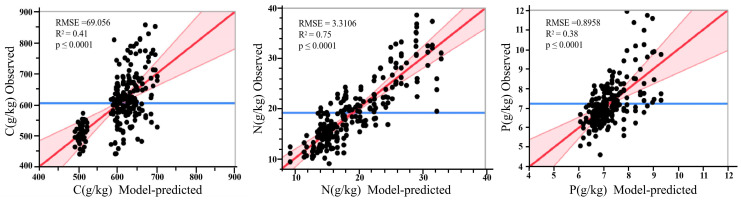
Summary of the observed vs. model-predicted C, N, and P nutrients in fescue leaves.

The fitted model highlights the impact and ranking of factors influencing the C, N, and P contents of five herbaceous forage leaves (Figure 4), in which C, N, and P nutrients served as the response variables. A significance level of *p* < 0.01 indicated a highly influential factor, with larger *p*-values corresponding to reduced impacts. As demonstrated in Figure 5, the model-predicted C, N, and P contents of forage leaves show an immensely significant correlation with the field-observed values (*p* < 0.01). The associated prediction models exhibited R^2^ values of 0.41, 0.75, and 0.38 for leaf C, N, and P, respectively. These outcomes underscore the varied influence exerted by distinct factors on the nutrient content of forage leaves. Moreover, all these factors and their secondary interactions significantly affect the C, N, and P contents of these leaves (*p* < 0.01). Thus, the joint application of sheep manure and granular organic fertilizer substantially impacts the nutrient content of forage leaves of different species. Consequently, we derived analytical formulas for predicting the C, N, and P contents of leaves of diverse forage types under varying dosages of sheep manure and granular organic fertilizer (Figure S1).

#### 3.3.3. Nutrient Changes with Organic Fertilizer Dosage

Utilizing the JMP PRO 16.0.0 statistical software, we predicted and characterized alterations in C, N, and P in forage leaves influenced by distinct variables. We sought to discern the trends in the impact of each variable on C, N, and P in forage leaves, ultimately identifying the optimal fertilization ratios. The aim was to maximize the C, N, and P contents of forage leaves within a specific range. After analyzing the aggregated effects of C, N, and P, we concluded that vegetation type, granular organic fertilizer, sheep manure, and their secondary interactions are exceptionally significant factors. Therefore, the dosages of sheep manure and granular organic fertilizer, along with shifts in vegetation type, are the paramount variables in the predictive model for the C, N, and P contents.

**Figure 6:**
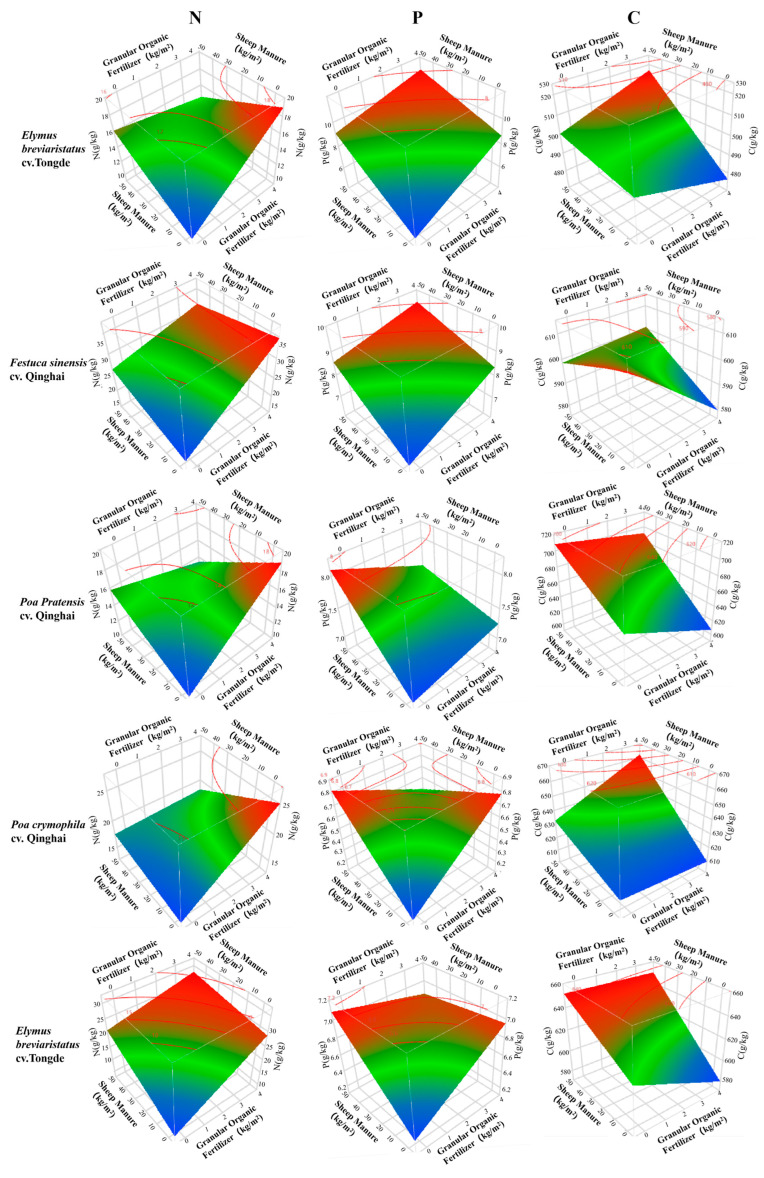
Curved surfaces of C, N, and P contents of leaves of different forage grasses at different dosages of sheep manure and granular organic fertilizer.

As depicted in Figure 6, the dosage of sheep manure exhibited a positive correlation with the C content for all five species of forage grass, while it was inversely correlated with the N content of *Elymus breviaristatus* cv. Tongde, *Poa crymophila* cv. Qinghai, *Puccinellia tenuiflora* cv. Qinghai, and *Poa pratensis* cv. Qinghai leaves. The N content of *Festuca sinensis* cv. Qinghai leaves diminished with increasing the sheep manure dosage. On the contrary, the P content in the leaves of the five forage grasses and the sheep manure had an opposite trend to that of the N content. As the dosage of granular organic fertilizer increased, the C content in *Elymus breviaristatus* cv. Tongde, *Poa crymophila* cv. Qinghai, and *Festuca sinensis* cv. Qinghai leaves gradually decreased. In *Poa pratensis* cv. Qinghai and *Puccinellia tenuiflora* cv. Qinghai leaves, the C content was positively correlated with the amount of granular organic fertilizer used. Meanwhile, the N and P contents in *Elymus breviaristatus* cv. Tongde and *Festuca sinensis* cv. Qinghai leaves escalated with an augmented granular organic fertilizer dosage. Conversely, this change in *Poa pratensis* cv. Qinghai and *Poa crymophila* cv. Qinghai leaves observed in the field ran counter to the previous findings. *Puccinellia tenuiflora* cv. Qinghai leaves exhibited positive and negative correlations with granular organic fertilizer dosage, affecting N and P contents, respectively.

The model aimed to “maximize” the C, N, and P contents of forage leaves. The overall willingness was determined as the geometric mean of individual response willingness functions, yielding values between 0 and 1. A higher overall willingness value indicated greater maximization of the C, N, and P contents of forage leaves at a specific fertilization rate. This also applied to the maximization of leaf C, N, and P contents for all the forage species under the given fertilizer ratio. Model predictions revealed *Elymus breviaristatus* cv. Tongde as the most successful performer among the five herbaceous species (Figure S2), boasting a willingness value of 0.57. The peak intention for the C, N, and P contents of *Elymus breviaristatus* cv. Tongde leaves occurred at a sheep manure dosage of 45 kg/m^2^ and a granular organic fertilizer dosage of 3.6 kg/m^2^. For *Elymus breviaristatus* cv. Tongde leaves, the predicted nutrient contents were 553.90–643.89 g/kg for C, 28.85–33.16 g/kg for N, and 8.72–9.89 g/kg for P at the 95% confidence intervals.

*Festuca sinensis* cv. Qinghai and *Poa pratensis* cv. Qinghai demonstrated willingness values of 0.48 and 0.30, respectively (Figures S3 and S4). Their leaf C, N, and P contents were maximized at the dosage of 3.6 kg/m^2^ of granular organic fertilizer and 45 kg/m^2^ of sheep manure. The nutrient contents of *Festuca sinensis* cv. Qinghai were predicted to be 599.05–689.04 g/kg for C, 24.71–29.02 g/kg for N, and 6.48–7.65 g/kg for P. For *Poa pratensis* cv. Qinghai, these values changed correspondingly to 477.14–567.14 g/kg for C, 12.74–17.05 g/kg for N, and 6.57–7.74 g/kg for P.

*Poa crymophila* cv. Qinghai exhibited a willingness value of 0.42 (Figure S5). The C, N, and P contents of its leaves displayed a negative correlation with granular organic fertilizer dosage. The optimal dosage was found to be 0 kg/m^2^ of granular organic fertilizer and 45 kg/m^2^ of sheep manure. The predicted contents were 656.28–746.27 g/kg for C, 13.01–17.32 g/kg for N, and 7.34–8.52 g/kg for P. For *Puccinellia tenuiflora* cv. Qinghai, the willingness value stood at 0.43 (Figure S6). The N and P contents of its leaves were negatively correlated with sheep manure dosage. The optimal ratio consisted of 3.6 kg/m^2^ of granular organic fertilizer and 0 kg/m^2^ of sheep manure.

## 4. Discussion

### 4.1. Effects of Fertilizer Dosage on Leaf Nutrient Content and Ecological Chemometrics

Plants rely on essential nutrients like carbon, nitrogen, and phosphorus for growth [27,28], and their equilibrium governs plant development [29]. Different plant types have varying abilities to absorb nutrients in terms of type, quantity, and efficiency. Our results reveal distinctions in the leaf C, N, and P contents of five herbaceous species at diverse fertilization levels. This divergence likely stems from varied adaptability to environmental shifts and distinct nutrient adaptation strategies [30]. Changes in organic fertilizer dosage minimally influence the leaf C content of the five forage species, a finding that is aligned with that of Zheng’s study on plant nutrient changes induced by bacterial-agent-infused organic fertilization [31]. This minimal impact on the C content may arise from its role as a structural component within plants, causing it to exhibit limited variations and reduced susceptibility to external influences [32]. Comparatively, the leaf N content of these five forage species was 19.24 g/kg, very close to the N content (23.5 g/kg) obtained from the Qinghai–Tibetan Plateau [33]. However, leaf P content measured at 7.22 g/kg significantly exceeds the levels observed in previous studies on the Qinghai–Tibetan Plateau (1.9 g/kg) and globally (1.8 g/kg) [34,35]. Consequently, the N/P ratio is lower in our study than in the studies of Yang and Yu et al. (13.9 and 15.7) [33,35]. Species with a higher N and P content and lower N/P ratio generally exhibit reduced internal stability [13], which is aligned with the stoichiometric internal stability theory [36] that links lower internal stability to decreased productivity and stability [37]. In this study, the trend of N/P changes in forage leaves is positively correlated with organic fertilizer dosage, suggesting that organic fertilizer plays a role in enhancing plant productivity and stability.

In most fertilization scenarios, the leaf N/P ratio was the highest in *Elymus breviaristatus* cv. Tongde among the five forage species. This implies that this species has superior productivity and stability among the five species. In terms of internal stability through N/P, the productivity and stability of the five species are ranked as follows: *Elymus breviaristatus* cv. Tongde > *Festuca sinensis* cv. Qinghai > *Poa pratensis* cv. Qinghai > *Poa crymophila* cv. Qinghai. However, in this study, the N/P ratio of the manually restored grassland in the frigid alpine mining region is notably lower than both the global level and the level observed in the natural grasslands of the Qinghai–Tibetan Plateau [38], creating a disparity in productivity and stability.

After studying vegetation adaptability in frigid alpine mining areas, Sun [39] found the N/P ratio of forage leaves to be 4.68 at a single granular organic fertilizer dosage of 2.5 kg/m^2^, which resulted in favorable vegetation characteristics. This value closely resembles the average N/P ratio of 3.21 observed in the forage leaves in the M3S1 treatment (2.4 kg/m^2^ granular organic fertilizer and 0 kg/m^2^ sheep manure) in this study. However, this value notably diverges from Koerselman et al.’s N/P threshold hypothesis [40] because of the distinct environmental conditions in the frigid alpine mining areas from the global contexts that introduce a potential distortion risk for the 14/16 threshold hypothesis [41]. This study’s deviation from the N/P threshold hypothesis leads to the conclusion that this method is not suitable for determining nutrient limitations in frigid alpine mining areas. Future research will have to rely on large-scale nutrient dynamics within “plants soil microorganisms” and the relative reabsorption theory [42] to determine nutrient limitations and discover universally applicable N/P thresholds for identifying limiting nutrients in these mining areas.

### 4.2. Vegetation Nutrients and Organic Fertilizer Dosage

Plant material production serves as a key carbon fixation pathway in ecosystems, with leaves playing a pivotal role in this process. An understanding of the linkage between leaf nutrient content and vegetation productivity holds significant importance in ecological research [43]. Nitrogen (N) and phosphorus (P) are common limiting elements in both terrestrial and aquatic ecosystems [44]. An elevated N content in plants within a certain range corresponds to increased soil N availability and plant productivity [45]. Yet, solely pursuing higher N and P contents in plants may disrupt the stoichiometric balance among elements in plant organs, leading to imbalanced relationships and reduced productivity, which are unfavorable for plant growth [46]. In our study, the mean C, N, and P contents in the leaves of the five forage species in the unfertilized conditions (M0S0) (607.13 g/kg, 13.452 g/kg, and 6.43 g/kg) were notably lower than in leaves undergoing alternative treatments in the fertilizer-reconstructed soil. Prior research has demonstrated that the use of organic fertilizers significantly boosts vegetation height, coverage, and aboveground biomass during the restoration of an alpine mining area (*p* < 0.05). The sole reliance on coal gangue substrate to restore the mining areas falls short of ecological restoration standards [10]. This underscores the correlation between elevated C, N, and P contents in plant leaves within a certain range and improved vegetation productivity and health. Our research reveals distinct responses of leaf C, N, and P contents across different herbaceous varieties to varying dosages of sheep manure and granular organic fertilizer. These differences could stem from varying functional and life strategies among different herbaceous types under diverse nutrient conditions, each requiring specific amounts and proportions of nutrients to sustain growth, resulting in distinct nutrient demands [47]. Take *Elymus breviaristatus* cv. Tongde as an example. As the granular organic fertilization level increases, its leaf C content decreases, while the N and P contents rise. This may result from the carbon secretion into the rhizosphere, nourishing the soil microbiota upon granular organic fertilizer application, leading to C consumption and the release of N and P for plant absorption. Furthermore, the proportion of C, N, and P contents in the organic fertilizers varies, leading to contrasting effects among different types of organic fertilizer. Certain organic fertilizers exhibit higher N and P contents, coupled with a lower C content. Their joint application leads to a decreased C content but increased N and P contents of leaves. Additionally, sheep manure contains relatively elevated N levels; a generous dosage raises soil N concentration, potentially hindering plant N absorption. This translates to a decreased N content in most grasses. Conversely, the sheep manure’s lower P content may lead to insufficient soil P concentration upon application, limiting plant P absorption. To counteract these effects, plants may elevate P absorption by increasing C absorption, resulting in augmented C and P contents within plants.

This study also highlighted *Elymus breviaristatus* as the top-performing herbaceous variety in characterizing the response variables of leaf C, N, and P contents, followed by *Elymus breviaristatus* cv. Tongde > *Festuca sinensis* cv. Qinghai > *Poa crymophila* cv. Qinghai > *Poa pratensis* cv. Qinghai. These findings are aligned with the productivity and stability ranking of the herbaceous species, as inferred from the actually measured vegetation N/P ratio, which mirrors *Elymus breviaristatus* cv. Tongde > *Festuca sinensis* cv. Qinghai > *Poa pratensis* cv. Qinghai > *Poa crymophila* cv. Qinghai. This suggests that the result of the overall maximum willingness has been soundly predicted for all three response variables (C, N, and P). Such insights are relevant to the actual ecological restoration in frigid alpine mining regions.

Through the least squares fitting model, this study has determined that cultivating *Elymus breviaristatus* cv. Tongde, *Festuca sinensis* cv. Qinghai, and *Poa pratensis* cv. Qinghai in pastures to maximize leaf C, N, and P contents requires the joint use of granular organic fertilizer and sheep manure at dosages of 3.6 kg/m^2^ and 45.0 kg/m^2^, respectively. This optimal ratio of fertilization, where both organic fertilizers reach their maximum application, can be attributed to the short study duration, reduced biological activity due to the mining area’s high altitude and cold climate, and the gradual decomposition of sheep manure, leading to slow nutrient release and plant utilization. Therefore, subsequent investigations should focus on fertilizer treatments, as they exhibit superior performance in meeting the nutrient demands of the five forage species, accounting for the varying degrees of decomposition of sheep manure.

Ecological restoration in mining areas necessitates a prolonged commitment [48]. In a study by Qiao [49] on organic fertilizer’s impact on soil physicochemical properties and microorganisms in frigid alpine mining areas, granular organic fertilizer and sheep manure were deemed indispensable for soil enhancement and mining area reconstruction. Our study uncovered negative effects of granular organic fertilizer or sheep manure on the leaf C, N, and P contents of *Poa crymophila* cv. Qinghai and *Puccinellia tenuiflora* cv. Qinghai. Consequently, future seeding efforts should be mindful of the adverse responses of leaf C, N, and P contents for certain species to the application of sheep manure or granular organic fertilizer in ecologically restoring alpine mining area, as evidenced by our findings on *Puccinellia tenuiflora* cv. Qinghai and *Poa crymophila* cv. Qinghai.

## 5. Conclusions

This study investigated the optimal blend of sheep manure with granular organic fertilizer to enhance C, N, and P nutrient levels in diverse forage leaves. The C, N, and P contents and the stoichiometric characteristics of five forage grasses (*Elymus breviaristatus* cv. Tongde, *Poa crymophila* cv. Qinghai, *Puccinellia tenuiflora* cv. Qinghai, *Festuca sinensis* cv. Qinghai, and *Poa pratensis* cv. Qinghai) in alpine mining area soil, which was reconstructed through organic fertilizers, exhibited varied responses to the application of granular organic fertilizer and sheep manure. Generally, as the applied organic fertilizer dosage increased, the leaf C, N, and P contents of the five forage grasses increased, significantly improving the N and P contents and N/P ratio (*p* < 0.05) of tame vegetation in the alpine mining area. The P content also showed a consistent upward trend with increased organic fertilizer dosage, leading to significantly higher N and P contents and a significantly higher N/P ratio of the manually restored grassland in the alpine mining area (*p* < 0.05). The optimal fertilizer dosage for *Elymus breviaristatus* cv. Tongde, *Festuca sinensis* cv. Qinghai, and *Poa pratensis* cv. Qinghai was found to be 3.6 kg/m^2^ of granular organic fertilizer and 45.0 kg/m^2^ of sheep manure. These findings hold theoretical significance for understanding the ecological mechanisms behind vegetation restoration in alpine mining areas and for developing optimal strategies for vegetation restoration in these areas.

## Figures and Tables

**Table 1:** Physical and chemical properties of the substrate of the test site amended with coal gangue, sheep manure, and granular organic fertilizers.

	Total Nitrogen (g/kg)	Total Phosphorus (g/kg)	Organic Matter (g/kg)	pH
Coal gangue substrate	2.24	0.51	62.50	7.32
Sheep manure	11.2	2.89	158	7.80
Granular organic fertilizer	34.5	4.69	450	7.72

**Table 2:** Treatments of the sampling plots with different dosages of granular organic fertilizers and sheep manure.

Treatments	Granular Organic Fertilizer (kg/m^2^)	Sheep Manure		
		Sheep Manure to Coal Gangue Ratio	Sheep Manure Volume (m^3^/m^2^)	Sheep Manure Dosage (kg/m^2^)
M0S0	0	0:10	0	0
M0S1	0	1.5:10	0.03	15
M0S2	0	3:10	0.06	30
M0S3	0	4.5:10	0.09	45
M1S0	1.2	0:10	0	0
M1S1	1.2	1.5:10	0.03	15
M1S2	1.2	3:10	0.06	30
M1S3	1.2	4.5:10	0.09	45
M2S0	2.4	0:10	0	0
M2S1	2.4	1.5:10	0.03	15
M2S2	2.4	3:10	0.06	30
M2S3	2.4	4.5:10	0.09	45
M3S0	3.6	0:10	0	0
M3S1	3.6	1.5:10	0.03	15
M3S2	3.6	3:10	0.06	30
M3S3	3.6	4.5:10	0.09	45

Note: M0, M1, M2, and M3 represent different sheep manure treatments; S0, S1, S2, and S3 represent different granular organic fertilizer treatments.

**Table 3:** Three-way ANOVA of vegetation species (V), granular organic fertilizer (G), and sheep manure dosage (S) on leaf C, N, and P contents and stoichiometric characteristics.

Items	C	N	P	C:N	C:P	N:P
V	**	**	**	**	**	**
G	ns	**	ns	**	*	**
S	*	**	**	ns	*	*
V × G	ns	**	ns	**	ns	**
V × S	ns	**	ns	**	*	**
G × S	ns	**	ns	**	ns	**
V × G × M	*	**	ns	**	*	**

Note: “ns” indicates no-significant difference, “*” the significant difference at *p* < 0.05, “**” the very significant difference at *p* < 0.001.

## Data Availability

Data are contained within the article and Supplementary Materials.

## Supplementary Materials

The following supporting information can be downloaded at https://www.mdpi.com/article/10.3390/plants12223838/s1, Figure S1: Analytical equation for predicting the C, N, and P contents of different products of tame forages under respective dosages of sheep manure and granular organic fertilizer; Figure S2: Willingness to maximize the C, N and P contents of *Elymus breviaristatus* cv. Tongde leaves and the predicted optimal fertilizer ratios; Figure S3: Willingness to maximize the C, N and P contents of *Festuca sinensis* cv. Qinghai leaves and the predicted optimal fertilizer ratios; Figure S4: Willingness to maximize the C, N and P contents of *Poa pratensis* cv. Qinghai leaves and prediction of optimal fertilization ratios; Figure S5: Willingness to maximize the C, N and P contents of *Poa crymophila* cv. Qinghai leaves and predicted optimal fertilizer ratios. Figure S6. Willingness to maximize the C, N and P contents of *Puccinellia tenuiflora* cv. Qinghai leaves and the predicted optimal fertilization ratios.
