# Supplementary material for: A Study on the C, N, and P Contents and Stoichiometric Characteristics of Forage Leaves Based on Fertilizer-Reconstructed Soil in an Alpine Mining Area
Source: Plants (Basel). 2023 Nov 13;12(22):3838. doi: 10.3390/plants12223838 (PMC10674538; doi:10.3390/plants12223838)
Supplement: Supplementary file 1 [file plants-12-03838-s001.zip › plants-2667518-supplementary.pdf]

**Supplementary Materials:**

$$Y(C) = 593.788 - 2.476 \times \text{GOF} + 0.680 \times \text{SM}$$

$$+ \text{Match}(\text{VT}) \begin{cases} 97.631(\textit{Poa Pratensis} \text{ cv. Qinghai}) \\ -7.497(\textit{Elymus breviaristatus} \text{ cv. Tongde}) \\ 61.019(\textit{Poa crymophila} \text{ cv. Qinghai}) \\ 24.825(\textit{Puccinellia tenuiflora} \text{ cv. Qinghai}) \\ 19.284(\textit{Festuca sinensis} \text{ cv. Qinghai}) \end{cases} + (\text{GOF} - 1.8) \times ((\text{SM} - 22.5) \times 0.139) + (\text{SM}$$

$$- 22.500)$$

$$\times \text{Match}(\text{VT}) \begin{cases} -0.249(\textit{Poa Pratensis} \text{ cv. Qinghai}) \\ -0.587(\textit{Elymus breviaristatus} \text{ cv. Tongde}) \\ 0.549(\textit{Poa crymophila} \text{ cv. Qinghai}) \\ 0.014(\textit{Puccinellia tenuiflora} \text{ cv. Qinghai}) \\ 0.301(\textit{Festuca sinensis} \text{ cv. Qinghai}) \end{cases} + (\text{GOF}$$

$$- 1.800) \times \text{Match} \begin{cases} 2.362(\textit{Poa Pratensis} \text{ cv. Qinghai}) \\ -0.846(\textit{Elymus breviaristatus} \text{ cv. Tongde}) \\ -5.083(\textit{Poa crymophila} \text{ cv. Qinghai}) \\ 5.306(\textit{Puccinellia tenuiflora} \text{ cv. Qinghai}) \\ -1.740(\textit{Festuca sinensis} \text{ cv. Qinghai}) \end{cases}$$

$$Y(N) = 15.688 + 1.550 \times \text{GOF} + 0.033 \times \text{SM}$$

$$+ \text{Match}(\text{VT}) \begin{cases} -4.267(\textit{Poa Pratensis} \text{ cv. Qinghai}) \\ 8.597(\textit{Elymus breviaristatus} \text{ cv. Tongde}) \\ -4.507(\textit{Poa crymophila} \text{ cv. Qinghai}) \\ 0.439(\textit{Puccinellia tenuiflora} \text{ cv. Qinghai}) \\ 0.617(\textit{Festuca sinensis} \text{ cv. Qinghai}) \end{cases} + (\text{GOF} - 1.8) \times ((\text{SM} - 22.500) \times (-0.042)) + (\text{SM}$$

$$- 22.5)$$

$$\times \text{Match}(\text{VT}) \begin{cases} -0.024(\textit{Poa Pratensis} \text{ cv. Qinghai}) \\ 0.005(\textit{Elymus breviaristatus} \text{ cv. Tongde}) \\ 0.040(\textit{Poa crymophila} \text{ cv. Qinghai}) \\ 0.067(\textit{Puccinellia tenuiflora} \text{ cv. Qinghai}) \\ 0.137(\textit{Festuca sinensis} \text{ cv. Qinghai}) \end{cases} + (\text{GOF} - 1.800) \times \text{Match} \begin{cases} -0.771(\textit{Poa Pratensis} \text{ cv. Qinghai}) \\ 0.788(\textit{Elymus breviaristatus} \text{ cv. Tongde}) \\ -0.923(\textit{Poa crymophila} \text{ cv. Qinghai}) \\ -0.247(\textit{Puccinellia tenuiflora} \text{ cv. Qinghai}) \\ 1.153(\textit{Festuca sinensis} \text{ cv. Qinghai}) \end{cases}$$

$$Y(P) = 6.743 + 0.077 \times \text{GOF} + 0.0150 \times \text{SM}$$

$$+ \text{Match}(\text{VT}) \begin{cases} -0.462(\textit{Poa Pratensis} \text{ cv. Qinghai}) \\ 1.011(\textit{Elymus breviaristatus} \text{ cv. Tongde}) \\ 0.223(\textit{Poa crymophila} \text{ cv. Qinghai}) \\ -0.547(\textit{Puccinellia tenuiflora} \text{ cv. Qinghai}) \\ -0.226(\textit{Festuca sinensis} \text{ cv. Qinghai}) \end{cases} + (\text{GOF} - 1.800) \times ((\text{SM} - 22.500) \times (-0.004)) + (\text{SM}$$

$$- 22.500)$$

$$\times \text{Match}(\text{VT}) \begin{cases} 0.008(\textit{Poa Pratensis} \text{ cv. Qinghai}) \\ 0.014(\textit{Elymus breviaristatus} \text{ cv. Tongde}) \\ -0.0020(\textit{Poa crymophila} \text{ cv. Qinghai}) \\ -0.121(\textit{Puccinellia tenuiflora} \text{ cv. Qinghai}) \\ -0.009(\textit{Festuca sinensis} \text{ cv. Qinghai}) \end{cases} + (\text{GOF}$$

$$- 1.800) \times \text{Match} \begin{cases} -0.063(\textit{Poa Pratensis} \text{ cv. Qinghai}) \\ 0.237(\textit{Elymus breviaristatus} \text{ cv. Tongde}) \\ -0.923(\textit{Poa crymophila} \text{ cv. Qinghai}) \\ -0.247(\textit{Puccinellia tenuiflora} \text{ cv. Qinghai}) \\ 1.153(\textit{Festuca sinensis} \text{ cv. Qinghai}) \end{cases}$$

**Figure S1.** Analytical equation for predicting the C, N, and P contents of different products of tame forages under respective dosages of sheep manure and granular organic fertilizer.

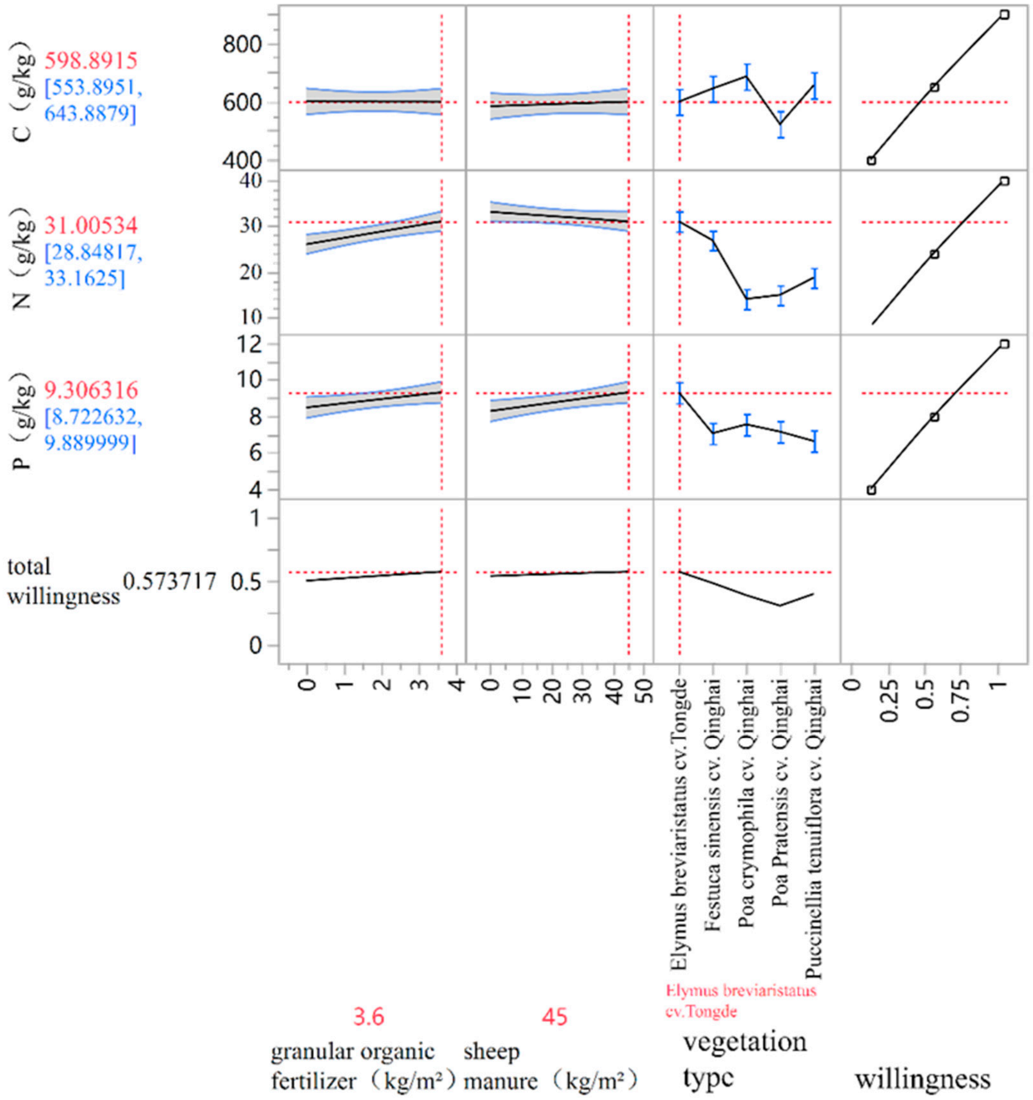


**Figure S2.** Willingness to maximize the C, N and P contents of *Elymus breviaristatus* cv. Tongde leaves and the predicted optimal fertilizer ratios.

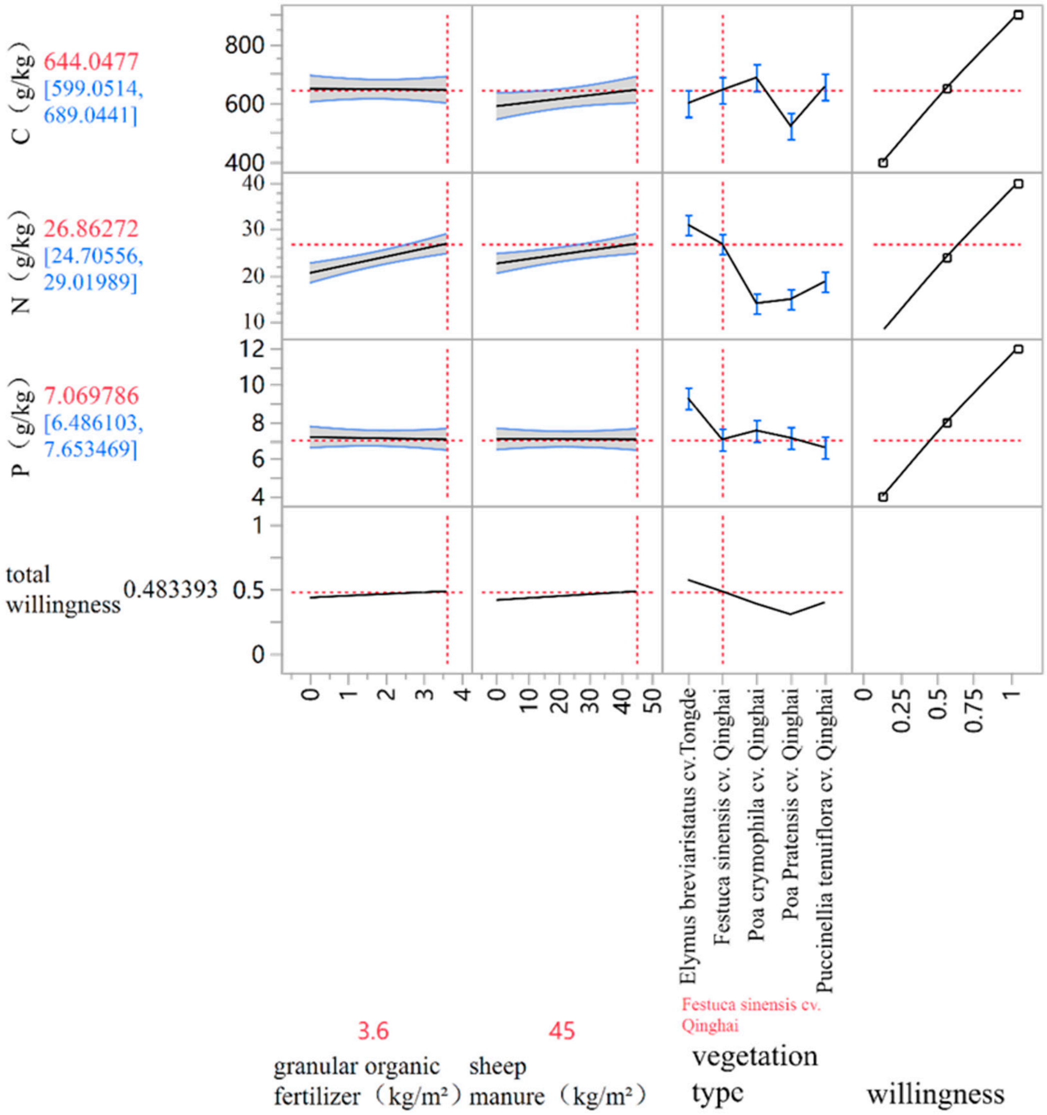


**Figure S3.** Willingness to maximize the C, N and P contents of *Festuca sinensis* cv. Qinghai leaves and the predicted optimal fertilizer ratios.

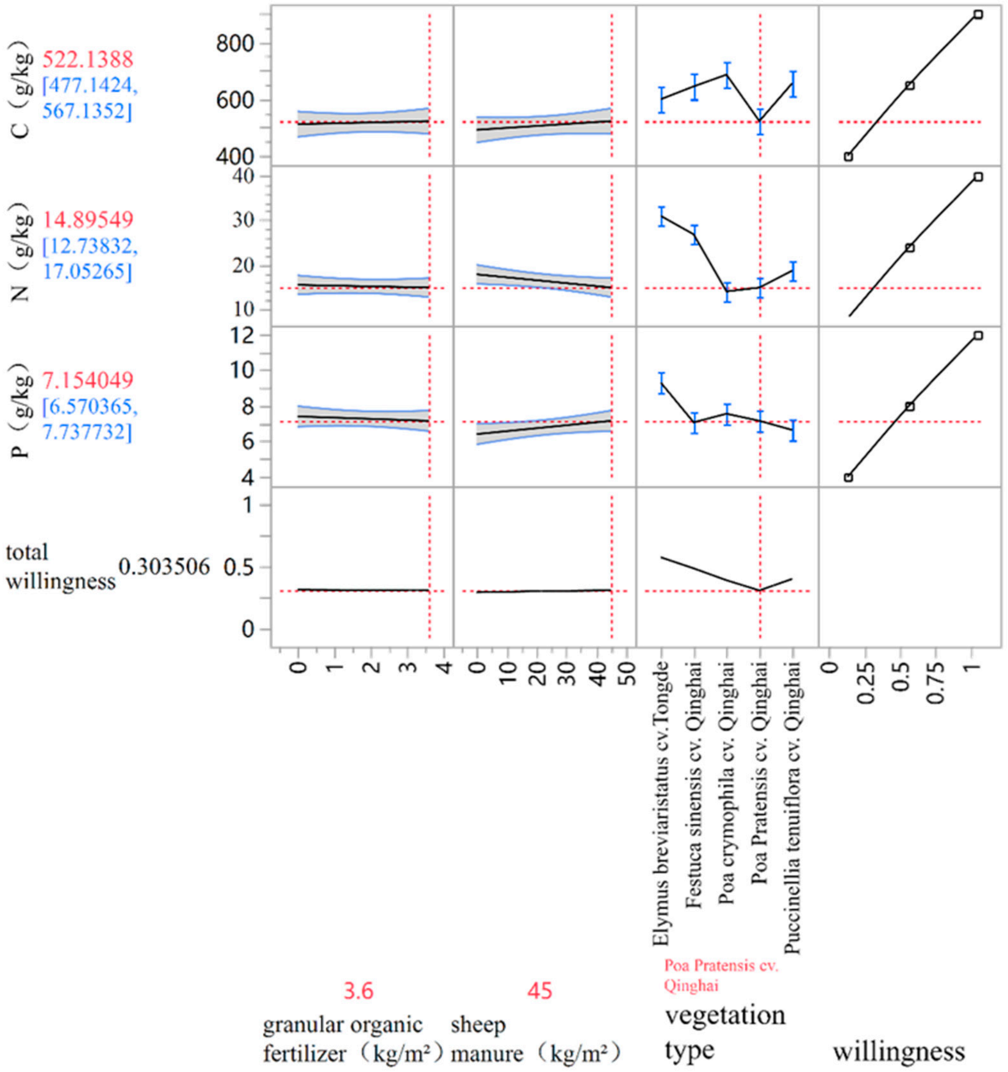


**Figure S4.** Willingness to maximize the C, N and P contents of *Poa Pratensis* cv. Qinghai leaves and prediction of optimal fertilization ratios.

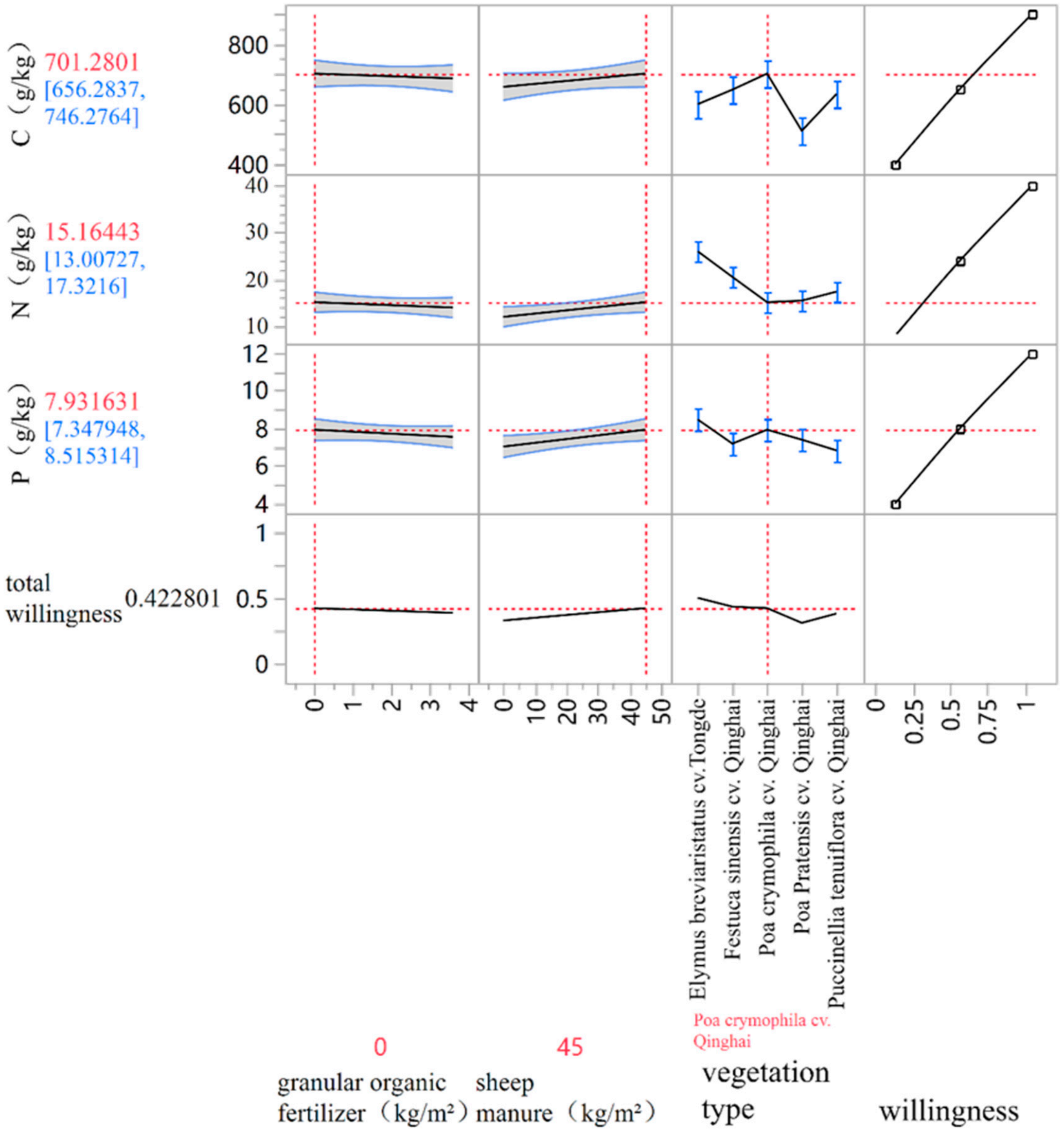


**Figure S5.** Willingness to maximize the C, N and P contents of *Poa crymophila* cv. Qinghai leaves and predicted optimal fertilizer ratios.

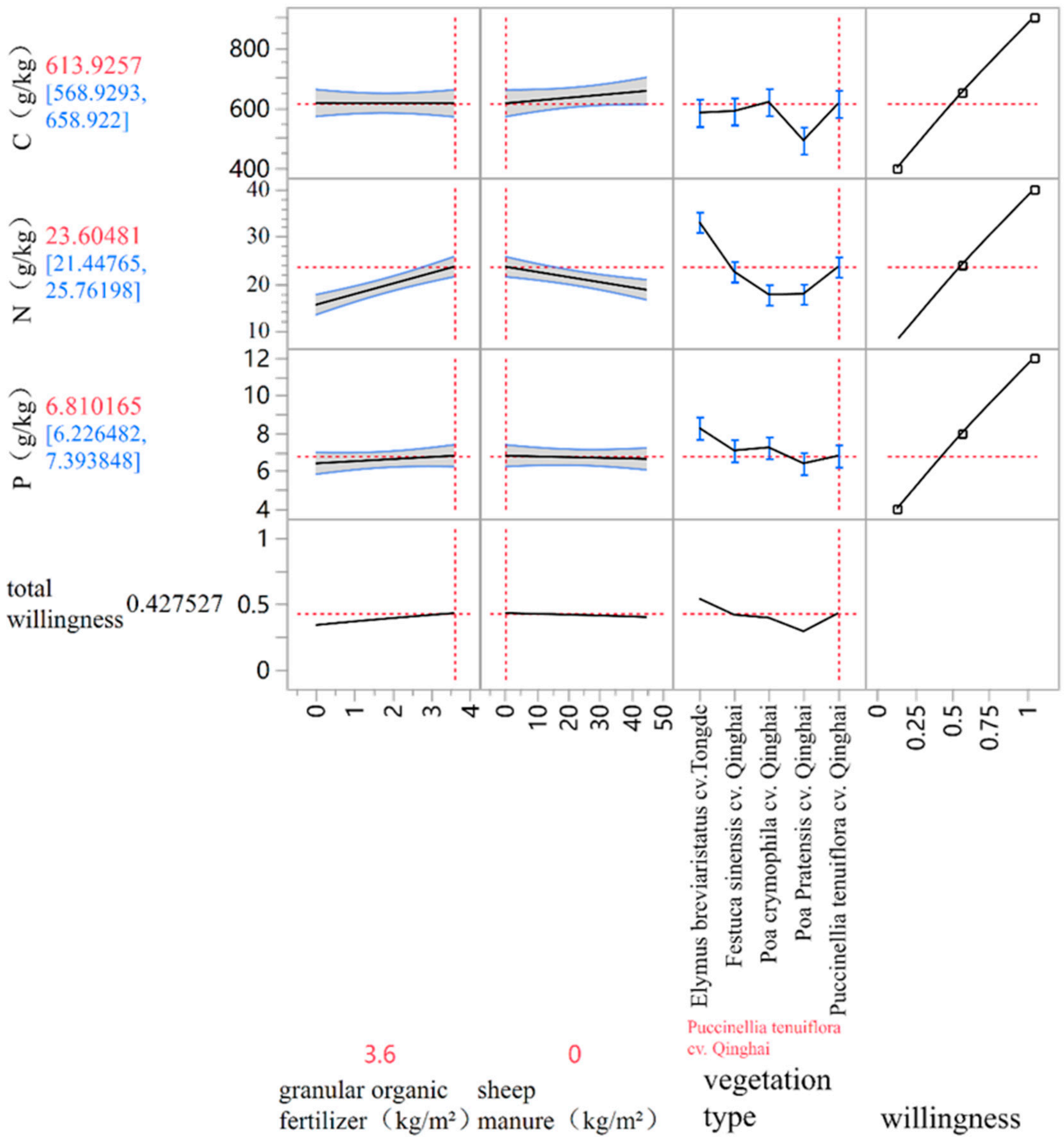


**Figure S6.** Willingness to maximize the C, N and P contents of *Puccinellia tenuiflora cv*. Qinghai leaves and the predicted optimal fertilization ratios.
